# Automatic Thalamus Segmentation from Magnetic Resonance Images Using Multiple Atlases Level Set Framework (MALSF)

**DOI:** 10.1038/s41598-017-04276-6

**Published:** 2017-06-27

**Authors:** Minghui Zhang, Zhentai Lu, Qianjin Feng, Yu Zhang

**Affiliations:** Guangdong Provincial Key Laboratory of Medical Image Processing, Southern Medical University, Guangzhou, 510515 China

## Abstract

In this paper, we present an original multiple atlases level set framework (MALSF) for automatic, accurate and robust thalamus segmentation in magnetic resonance images (MRI). The contributions of the MALSF method are twofold. First, the main technical contribution is a novel label fusion strategy in the level set framework. Label fusion is achieved by seeking an optimal level set function that minimizes energy functional with three terms: label fusion term, image based term, and regularization term. This strategy integrates shape prior, image information and the regularity of the thalamus. Second, we use propagated labels from multiple registration methods with different parameters to take full advantage of the complementary information of different registration methods. Since different registration methods and different atlases can yield complementary information, multiple registration and multiple atlases can be incorporated into the level set framework to improve the segmentation performance. Experiments have shown that the MALSF method can improve the segmentation accuracy for the thalamus. Compared to ground truth segmentation, the mean Dice metrics of our method are 0.9239 and 0.9200 for left and right thalamus.

## Introduction

**Figure 1:**
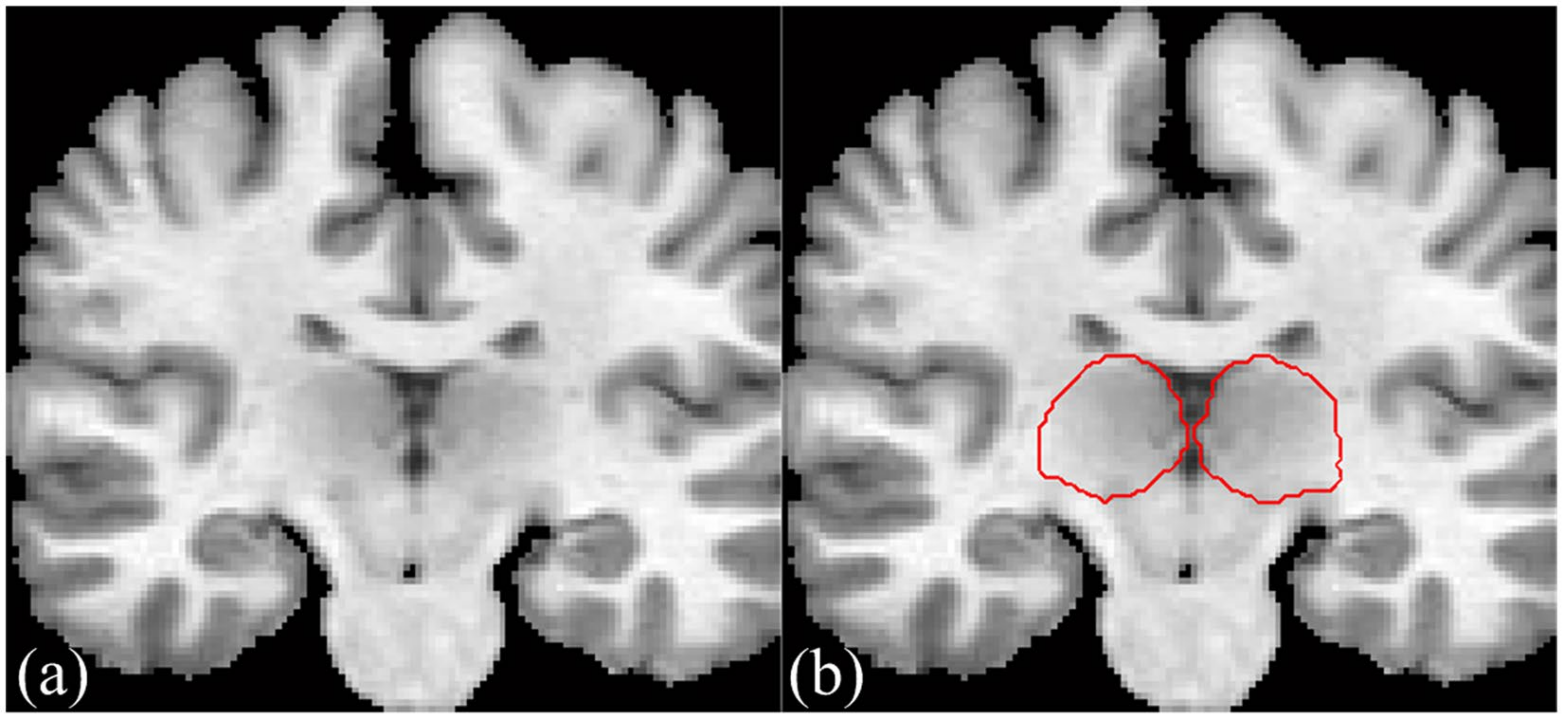
MR T1-weighted image (**a**) an image slice obtained from a 3-D thalamus MR image. (**b**) The manually-delineated thalamus boundary shown by the red contour, superimposed on the original image in (**a**).

The thalamus plays a critical role in human brain function. It is the largest, most internal structure of the diencephalon. Segmentation and characterization of the thalamus from brain magnetic resonance images (MRI) are expected to help noninvasive diagnosis and treatment. Thalamus segmentation has become more and more important for a wide range of clinical and research applications. For example, the thalamus changes in terms of volume and intensity are involved in a large number of diseases, such as Parkinson’s disease, multiple sclerosis and schizophrenia. Expert manual segmentation of thalamus from MRI data is still the gold standard, but it is labor intensive, and the segmentation results are not reproducible. The thalamus has no clearly contrasted boundaries in MR T1-weighted images, this issue is illustrated by Fig. 1, where no obvious thalamus boundary can be observed. It is a difficult challenge even for an expert radiologist.

Because of low image contrast, noise, and missing or diffuse boundaries, some discrete methods such as thresholding, FCM^1^, and region growing^2^ are not reliable because they only use image intensity information. Erhard *et al*. compared the performances of publicly available segmentation tools (volBrain, FSL, FreeSurfer and SPM) and their impact on diffusion quantification, emphasizing the importance of using recently developed segmentation algorithms and imaging techniques. They found that volBrain is superior in thalamus and hippocampus segmentation compared to FSL, FreeSurfer and SPM^3^. Shape is important information for brain structure segmentation. It is preferable to combine the intensity information along with the shape prior information. Active contours model (ACM) is a very popular technique for image segmentation which can combine shape prior information. According to the features of the involved image, ACM can be categorized as the image intensities statistical information (region-based ACM)^4–8^ or the image gradients (edge-based ACM)^9–12^. ACM needs precise initialization of the starting contours. It can not converge to the target structures unless the initialization is good enough. Another concern is that most of the boundaries of the thalamus are diffuse or missing, and thus it is difficult to segment the thalamus, even by a radiology expert. The performance of ACM may be adversely affected when these methods are employed. Shiyan Hu *et al*. combined levelset shape modeling and appearance modeling to identify the hippocampus and amygdala using multi-contrast MR imaging. The linear model for the gray-intensity of each contrast MR image can be constructed by applying principle component analysis (PCA) to the pre-processed gray images. PCA-based techniques require that the structures to be segmented are pre-aligned well^13^. Dinggang Shen *et al*. presented a learning-based algorithm by taking advantage of the multi-atlas framework and the auto-context model. Under the multi-atlas framework, auto-context model based classifiers are trained for all atlases to incorporate anatomical variability. It is time-consuming to extract image appearance features, texture features and context features. A sequence classifier is trained in each atlas space by borrowing the training samples from not only the underlying atlas but also all other linearly-aligned atlases, which has high computational complexity^14^.

Another popular methodology is atlas-based methods^15–17^. In these methods, the atlas includes a gray level image and a manually labelled image associated with the training image. The training gray level image is registered to the target image. The manually labelled images are thus propagated to the target image by using the deformation fields determined by registration method. The segmentation is accomplished with the help of image registration. The sophisticated non-rigid registration techniques can be used in a meticulous way, in which the shape prior and spatial information can be incorporated to help segmentation, so the atlas-based segmentation can be highly accurate.

Compared to other generic segmentation techniques, the atlas-based approach has several major advantages. For example, only a registration method and a number of pre-segmented data sets are required. There is no need for complex training procedures. The quality of the atlas-based approach is limited by the accuracy of the registration procedure and the anatomical similarity between the labelled and target image. Rather than depending on a single atlas, an alternative strategy is to register multiple atlas images to the target image separately^15^. These pairwise transformations are then used to propagate the labels to the target image. The final segmentation is achieved by fusing the propagated labels. A number of label fusion strategies have been proposed for multi-atlas based segmentation in the literature. Among them, the majority voting (MV) method is probably the simplest one and has been widely used in medical image segmentation^17^. In the MV method, the weights of candidate segmentations from each atlas are equal. The label with largest agreement from all atlases is assigned as the final label. A natural extension of the MV method is to use adaptive weighted averaging. Another popular approach is simultaneous truth and performance level estimation (STAPLE), which uses the expectation-maximization (EM) algorithm to achieve the best possible final segmentation^18^.

This multi-atlas based segmentation approach reduces the effect of errors associated with individually propagated atlases. A registration error for a particular propagated atlas is less likely to affect the final segmentation when combined with other atlases. Multi-atlas based segmentation methods have been proven to be effective and accurate in comparison with single atlas-based approaches. The quality of the registration algorithm and the selection of the atlas will directly affect the segmentation performance. The main limitations of the multi-atlas segmentation methods are that they often lead to a compromise between the accuracy of the registration and the smoothness of the deformation, and that those methods are time consuming.

To combine the advantages of the methods described above and overcome some of their disadvantages, we propose a new multiple atlas segmentation strategy using different registration methods and a level set fusion framework. The main contributions of this paper include the following. (1) The proposed method employs level set model further to improve the segmentation performance, which is able to provide smooth and closed contours in the final segmentation. (2) We explore the effect of the quality of registration method on segmentation accuracy. Multiple registration methods, i.e., ANTS^19^, DRAMMS^20^ and Demons^5^ are incorporated into the segmentation procedure, which facilitates combining the advantages of the different registration approaches. (3) The local similarity maps that calculated in the registration procedure are used as local weight of the level set function. This strategy does not add a substantial computational burden to the algorithm, since the atlases are registered to the target image offline. (4) The algorithm combines local intensity information, atlas prior, and shape regularity, it is very robust and able to accurately segment a new subject.

## Method

**Figure 2:**
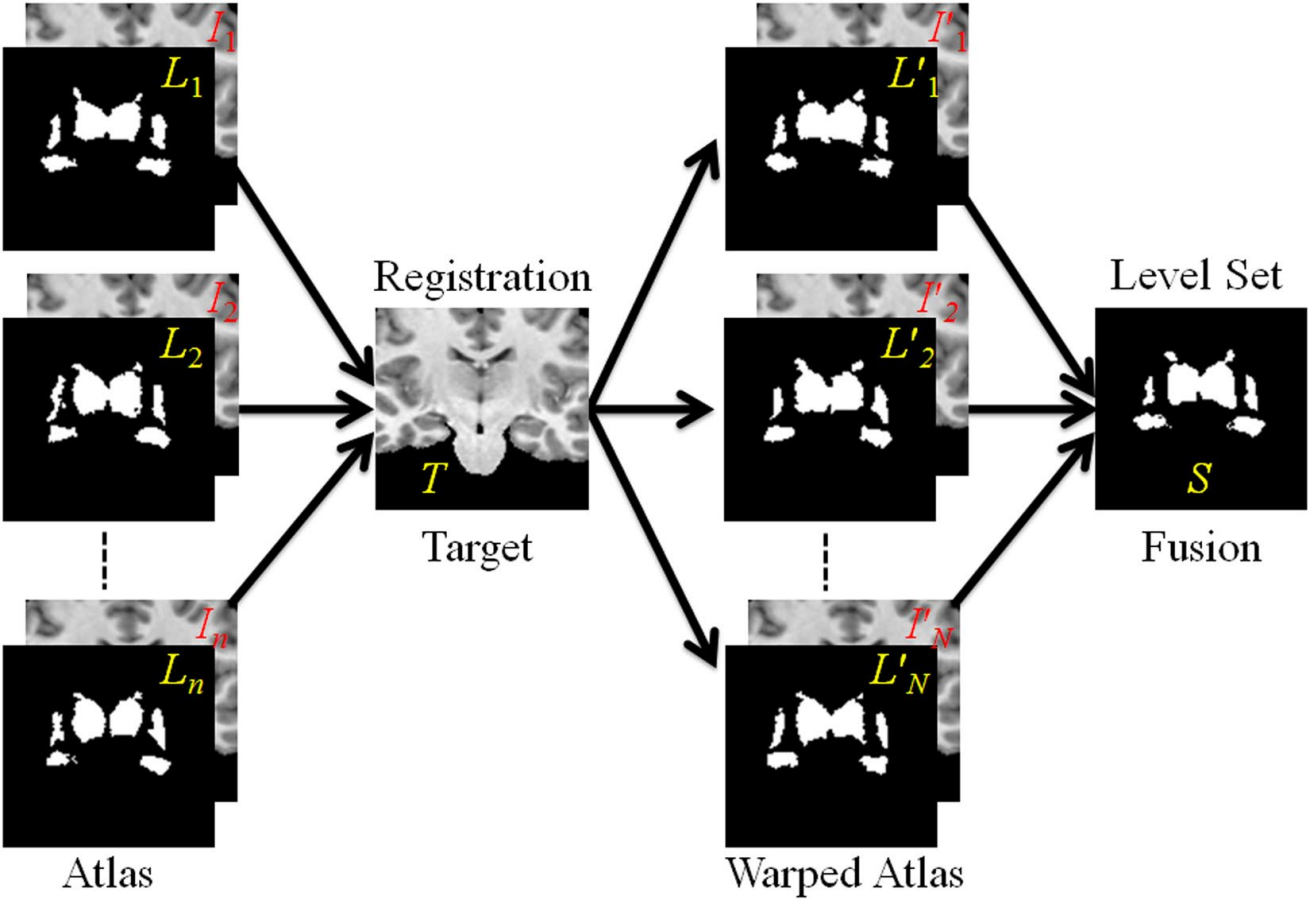
Schematic illustration of our segmentation method.

The proposed new segmentation method has two stages, multiple atlas registration (blue rectangle) and active contour model label fusion (red rectangle). It is illustrated schematically in Fig. 2. The gray level images *I*_*i*_ form the atlas is registered to the target image *T* by using ANTS, DRAMMS and Demons. The resulting transformations are used to warp the corresponding atlas label *L*_*i*_. The propagated label *L*′_*i*_ are then combined to create the final segmentation *S* of the target image by using the level set method.

### Ethics Statements

The original MRI scans were obtained from the Open Access Series of Imaging Studies (OASIS) project web site (http://www.oasis-brains.org/). OASIS is a project aimed at making MRI data sets of the brain freely available to the scientific community. Patient’s records and information were anonymized and de-identified prior to analysis. A subset of this data was used as the “gold standard” for the MICCAI 2013 Grand Challenge and Workshop on Multi-Atlas Labeling. Our study was approved by the ethics committee of Washington University Alzheimer’s Disease Research Center.

### Multiple Atlas Registration

Sources of error in atlas-based segmentations include registration error and manually delineated errors. The more accurately the registration warps the atlas onto the target image, the more accurate the result of the segmentation. The registration errors produced by using different atlases and different registration methods are not identical, and thus employing multiple atlases and multiple registration methods can effectively reduce the registration errors. To improve the segmentation performance of multiple atlas-based methods, we utilize many of the existing complementary registration strategies, i.e., ANTS^19^, and DRAMMS^20^.

Elastic registration methods such as statistical parametric mapping (SPM)^21^, free-form deformations (FFD)^22^, and Demons operate^23^ in the space of vector fields, which do not preserve topology. Not applying ad hoc constraints will result in the brain topology changing in an uncontrolled way. ANTS uses diffeomorphic transformations to warp images, and combines multiple different similarity measures that are optimized in parallel, which provide well-behaved solutions with mathematical guarantees about distance in deformation space and regularity.

DRAMMS bridges the gap between the traditional voxel-wise methods and landmark/feature-based methods. It renders each voxel relatively distinctively identifiable by a rich set of attributes to reduce match ambiguities. Additionally, DRAMMS modulates the registration by assigning higher weights to those voxels which have a higher ability to establish unique correspondences across images, therefore reducing the negative impact of those regions that are less capable of finding correspondences.

**Figure 3:**
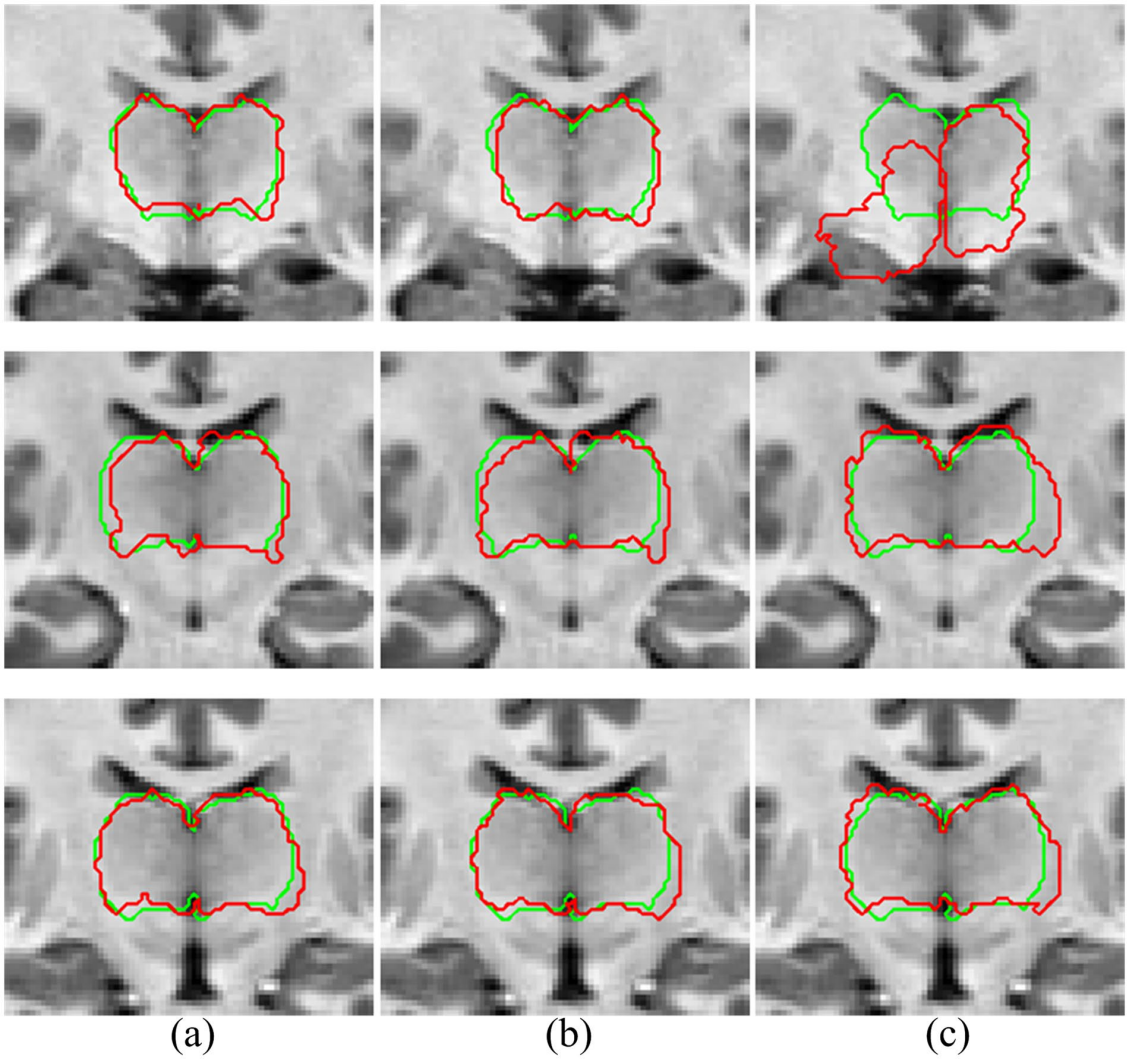
Registration performance comparison between ANTS, DRAMMS and Demons. (**a**) ANTS, (**b**) DRAMMS, (**c**) Demons. Each row represents different patient. The red contours in each column represent the warped thalamus boundary after ANTS, DRAMMS and Demons registration, respectively. The green contours are the ground truth that manually delineated by experts.

As shown in Fig. 3, ANTS and DRAMMS obviously outperform Demons. The performances of ANTS and DRAMMS are very similar, and it is difficult to evaluate which one is better, even by experts. To avoid possible registration error and to take full advantage of the merit of different registration methods, we combine the ANTS and DRAMMS registration results in a level set framework.

### Level Set Label Fusion

**Figure 4:**
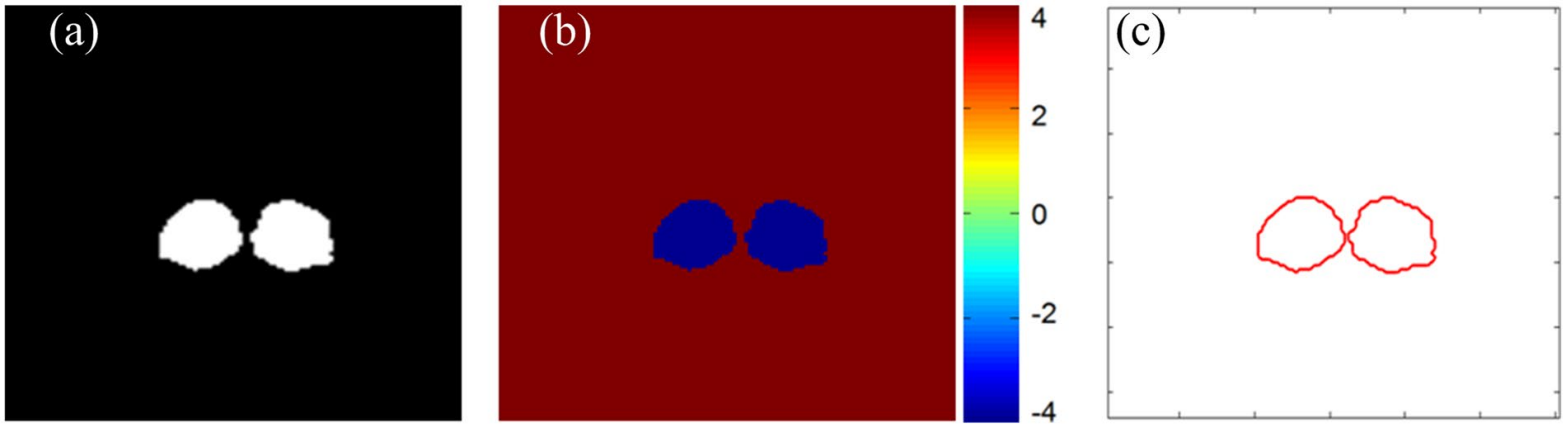
2D slice of an example of the shape modeling using the level-set method. (**a**) Manually segmented shape, (**b**) Level set function (LSF), (**c**) Zeros level set.

We use the level set method^8^ to model the structures of thalamus in the training data. First, the level set function (LSF) is utilized to represent the shape of manually segmented labels. Figure 4 shows the shape of the thalamus (showed in Fig. 1) by using the level set method, where the manually segmented thalamus is described by the LSF, and its boundary is captured by its zero level-set.

For a training data set with *n* labels, as we use ANTS and DRAMMS to register the atlases to target image, there are *N* = 2*n* separate LSF $\{{f}_{1},\ldots ,{f}_{N}\}$ where negative distances assigned to the inside and positive distances to the outside of object, are adopted to describe the boundaries of atlas shapes. The zero level contour of LSF *ϕ* is the boundary of the thalamus in the target image, denoted by *C*. Our purpose is to find an optimal LSF *ϕ* by using a label fusion technique under the proposed variational framework.

We propose a new and general variational level set formulation for label fusion, in which the energy *E* of LSF *ϕ* is defined in a general form:

1 $${E}(\varphi )=\alpha {F}(\varphi ;\,{\varphi }_{1},\ldots ,{\varphi }_{{N}})+\beta {D}(\varphi ;{I})+\gamma {R}(\varphi )$$

where F(*ϕ*; *ϕ*_1_, …, *ϕ*_*N*_) is the label fusion term, which can integrate the shape priors of different atlases; *D*(*ϕ*; *I*) is the image based term with a given target image *I*, which will combine the regional intensity information of the target image; and *R*(*ϕ*) is the regularization term that will smooth the shape of the thalamus in the level set evolution procedure. *α*, *β* and *γ* are the corresponding coefficients.

The label fusion term *F*(*ϕ*; *ϕ*_1_, …, *ϕ*_*N*_) is defined by

2 $${F}(\varphi ;\,{\varphi }_{1},\mathrm{...},{\varphi }_{N})={\int }_{{\rm{\Omega }}}{\sum }_{i=1}^{N}{\omega }_{i}(x)|\varphi (x)-{\varphi }_{i}(x){|}^{2}dx$$

where *ω*_*i*_(*x*) is a local weight assigned to the *i*-th label represented by the level set function *ϕ*_*i*_, with ${\sum }_{i=1}^{N}{\omega }_{i}(x)=1$, *x* indexes the image pixels, and Ω denotes the target image domain. Both ANTS and DRAMMS calculate the local similarity of each voxel in the registration procedure, where we use the local similarity as the local weight of the level set function. This strategy does not add computational burden to the algorithm, since the atlases are registered to the target image offline.

Minimizing label fusion energy *F*, the zero LSF *ϕ* is forced to be close to the zero LSF *ϕ*_1_, …, *ϕ*_*N*_, which are the boundaries of the thalamus given by the warped labels ${L^{\prime} }_{1},{L^{\prime} }_{2},\mathrm{...},{L^{\prime} }_{N}$. However, minimizing the energy *F* alone may result in an irregular shape of the contour obtained. Therefore, we need a regularization mechanism to maintain the regularity of the contour. We use the arc length of the zero level contour of the LSF *ϕ* as the regularization.

3 $${R}(\varphi )=\int |\nabla H(\varphi (x))|dx$$

This regularization term ensures the regularity of the boundary of the thalamus.

The traditional fusion method only combines the warped labels, without taking into account the target image information. These methods cannot correct the registration error and manually delineated errors effectively. We use the region-scalable fitting (RSF) model^24^ as the image term, which is able to handle intensity variations within the foreground and background of the image due to its localization property. This image term can guide the motion of the contour toward the thalamus boundaries, if there are some registration errors and manual labelling errors.

Given a point *y*, consider it’s neighborhood *O*_*y*_ = {*x*:|*x* − *y*|≤*ρ*}, which is divided by an object boundary *C* into two parts: *O*_*y*_∩*inside*(*C*) and *O*_*y*_∩*outside*(*C*). The image intensities *I*(*x*) in *O*_*y*_∩*inside*(*C*) and *O*_*y*_∩*outside*(*C*) can be approximated by the two constants *f*_1_(*y*) and *f*_2_(*y*), respectively. We seek an optimal contour *C* and fitting functions *f*_1_ and *f*_2_ such that the following energy

4 $${E}_{y}(C,\,{f}_{1}(y),\,{f}_{2}(y);\,I)={\lambda }_{1}{{\int }_{{O}_{y}\cap inside(C)}|I(x)-{f}_{1}(y)|}^{2}dx+{\lambda }_{2}{{\int }_{{O}_{y}\cap outside(C)}|I(x)-{f}_{2}(y)|}^{2}dx$$

is minimized for all *y* ∈ Ω for a given target image *I*, where *λ*_1_ and *λ*_2_ are the weighting coefficients. Minimization of *E*_*y*_(*C*, *f*_1_(*y*), *f*_2_(*y*); *I*) for all *y* can be achieved by minimizing the integration of *E*_*y*_ with respect to *y*, i.e., the energy *D* is defined by

5 $$D(C,\,{f}_{1},\,{f}_{2};\,I)={\int }_{{\rm{\Omega }}}{E}_{y}(C,\,{f}_{1}(y),\,{f}_{2}(y);\,I)dy$$

The energy *D*(*C*, *f*_1_, *f*_2_; *I*) can be expressed in an equivalent form in terms of LSF *ϕ* and the fitting functions *f*_1_ and *f*_2_ as the following:

6 $$D(C,\,{f}_{1},\,{f}_{2};\,I)={\sum }_{k=1}^{2}\int (\int {K}_{\rho }(x-y){|I(x)-{f}_{i}(y)|}^{2}{M}_{i}(\varphi (x))dx)dy$$

where *M*_1_(*ϕ*(*x*))=1−*H*(*ϕ*(*x*)) and *M*_2_(*ϕ*(*x*))=*H*(*ϕ*(*x*)), and where *H* is the Heaviside function. We approximate this as follows:

7 $${H}_{\varepsilon }(z)=\frac{1}{2}[1+\frac{2}{\pi }\arctan (\frac{z}{\varepsilon })]$$

where *K*_*ρ*_ is the kernel function. In this paper we chose a Gaussian kernel:

8 $${K}_{\rho }(u)=\frac{1}{{(2\pi )}^{n/2}{\rho }^{n}}{e}^{-{|u|}^{2}/2{\rho }^{2}}$$

with a scale parameter *ρ* > 0.

The above energy can be further expressed as

9 $$D(C,\,{f}_{1},\,{f}_{2};\,I)={\sum }_{k=1}^{2}\int (\int {K}_{\rho }(x-y){|I(x)-{f}_{i}(y)|}^{2}dy){M}_{i}(\varphi (x))dx$$

which defines a specific form of the image based term in the proposed variation framework. The minimization of the energy *E* with respect to the LSF can be achieved by using the standard gradient descent method^8^:

10 $$\frac{\partial \varphi }{\partial t}=-{\delta }_{\varepsilon }(\varphi )({\lambda }_{1}{e}_{1}-{\lambda }_{2}{e}_{2})+\upsilon {\delta }_{\varepsilon }(\varphi )div(\frac{\nabla \varphi }{|\nabla \varphi |})+\mu ({\nabla }^{2}\varphi -div(\frac{\nabla \varphi }{|\nabla \varphi |}))$$

where *δ*_*ε*_ is the smoothed Dirac delta function, and *e*_1_ and *e*_2_ are the functions

11 $${e}_{i}(x)=\int {K}_{\rho }(x-y){|I(x)-{f}_{i}(y)|}^{2}dy,\,i=1,2$$

## Parameter optimization

The parameter values of our proposed variational framework were chosen based on the 20 labeled images that were used for training. This was done by performing leave-one-out parameter-tuning experiments on those 20 images. For example, consider the case where image 1 was used as a target, and images 2 to 20 were used for training. To determine the optimal values of *α*, *β*, *γ*, *μ*, *v*, *λ*_1_, and *λ*_2_, 20 parameter-tuning experiments were conducted. First, the manual labels of images 2–20 were warped to the coordinate framework of image 1, and segmentation was performed using certain values of the parameters. The manual labels of image 1 were then used as the gold standard to compute the similarity index (*SI*) for these parameters. This was repeated for several values of *α*, *β*, *γ*, *μ*, *v*, *λ*_1_, and *λ*_2_. Similar parameter-tuning experiments were then performed for images 2–20, each yielding a list of *SI*-parameter combinations. The parameters that gave the highest *SI* averaged over all parameter-tuning experiments were chosen. We fixed the parameters for all experiments in our proposed method, as listed in Table 1.

**Table 1:** The parameters used in all experiments.

parameter	*α*	*β*	*γ*	*μ*	*v*	*λ*_1_	*λ*_2_
value	0.1	1	0.1	0.1	0.01	0.0001	0.0001

## Experiments results

In this section, several experiments were conducted to evaluate the proposed method. Since our method aims to improve segmentation accuracy in two ways, by incorporating multiple registration method into the conventional multiple atlas-based methods and by taking the advantage of level set framework, it is worthwhile to evaluate the contributions from these two different methods. First, we compared the proposed multiple registration method to label fusion using a single registration method. Second, we compared the proposed method to five state-of-the-art automatic segmentation methods: STAPLE^25^, Spatial STAPLE^26^, Major Voting^27^, Weight Voting^28^, and SIMPLE^29^. We use MASI Label Fusion software tools (http://www.nitrc.org/projects/masi-fusion). There are no parameters in Major Voting and Weight Voting, so we obtain the fusion results directly. For STAPLE, Spatial STAPLE, and SIMPLE, we use leave-one-out parameter-tuning experiments to get the best results, and then compare these with our proposed method. The Matlab source code for the Multiple Atlases Level Set Framework (MALSF) can be downloaded at https://github.com/luzhentai/MALSF.2.0.

## Data and Experiments set

Thirty-five real brain MRI images were downloaded from https://masi.vuse.vanderbilt.edu/. This web site provides the online continuation of a segmentation contest held at the 2013 Medical Image Computing and Computer Assisted Intervention Challenge (MICCAI). The dataset consists of a de-faced T1-weighted structural MRI and an associated manually labeled volume with one label per voxel. Each volume (MRI and label) will be stored in a separate 3D NiFTI file. The dimensions are 256 × 256 × 287, and the voxel dimensions are 1 × 1 × 1 mm. We randomly select fifteen images as targets and twenty images as training images.

All MR volumes are corrected for intensity inhomogeneity using the N4 bias field correction algorithm^30^. The intensities are then linearly normalized to the range of 0 to 255. The formula for the normalization is: $\frac{I-{I}_{\min }}{{I}_{\max }-{I}_{\min }}\times 255$, where *I*_min_ and *I*_max_ are the minimum and maximum intensity values in the volume, respectively.

To reduce computational burden, the segmentation is estimated only within the region of interest (ROI) surrounding the left and right thalamus. We use a very fast and simple approach that utilizes the union of all expert labels in the training database as the ROI. In this way, we ensure that the structure is completely included in the ROI.

## Quantitative evaluation of thalamus segmentation

To quantify the quality of our automatic segmentations, as well as of other state-of-the-art segmentation methods, we employed four widely used metrics, similarity index (*SI*), recall (*R*), relative overlap (*RO*), and precision (*P*), to measure the volumetric overlap of automatic segmentation with respect to the manual labels (i.e., ground truth). Moreover, we also measure the surface distance based on the Hausdorff distance (*HD*)^28^:

12 $$\begin{array}{lllll}{SI}=\frac{2V(A\cap B)}{V(A)+V(B)} & P=\frac{V(A\cap B)}{V(B)} & R=\frac{V(A\cap B)}{V(A)} & RO=\frac{V(A\cap B)}{V(A\cup B)} & HD=\,\max ({H}_{1},{H}_{2})\end{array}$$

where *V*(*A*) denotes the volume of the ground-truth segmentation, *V*(*B*) denotes the volume of automatic segmentation, ${H}_{1}={\max }_{a\in A}({\min }_{b\in B}d(a,b))$, ${H}_{2}={\max }_{b\in B}({\min }_{a\in A}d(a,b))$, an*d d*(*a*, *b*) is the Euclidean distance between two points *a* and *b*.

### Effect of using multiple registration method

We performed segmentation on the MICCAI data using the following method:

- *LS*-*ANTS:* our level set based fusion strategy that uses a single ANTS registration method
- *LS*-*DRAMMS:* our level set based fusion strategy that uses a single DRAMMS registration method
- *Our:* our level set based fusion strategy that uses multiple registration methods (ANTS and DRAMMS)

**Figure 5:**
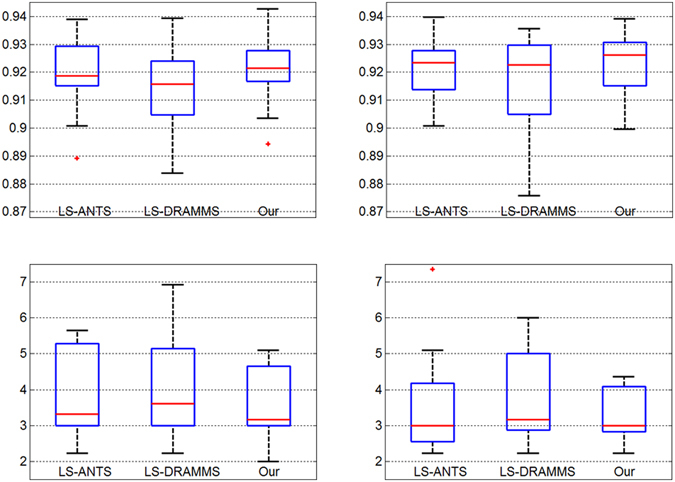
Box-plot of performance metric on the MICCAI dataset. The left column is left thalamus and the right column is right thalamus. The top row contains the similarity index, higher values implying greater volumetric overlap with the manual segmentation, and the bottom row contains the Hausdorff distance, lower values implying a surface closer to the manual segmentation.

Figure 5 shows summary box-plots of the similarity index (*SI*) and Hausdorff distance (*HD*) versus the manual delineate for each segmentation method on the MICCAI dataset. In top row, the red line is the median *SI* of fifteen test images, with higher values implying greater volumetric overlap with the manual segmentation. In bottom row, the red line is median Hausdorff distance, with lower values implying a surface closer to the manual segmentation. The results show that our method using multiple registrations methods is better than the one using a single registration method. The use of the multiple registrations captures greater anatomical variability and improves robustness against occasional registration failures.

### Effect of using the level set fusion method

To compare our results with those of recent automatic segmentation methods reported in the literature, we performed segmentation on the MICCAI data using several state-of-the-art fusion algorithms: STAPLE^25^, Spatial STAPLE^26^, Major Voting^27^, Weight Voting^28^, and SIMPLE^29^.

Table 2 shows the mean values and standard deviations of the similarity index for the MICCAI data. The mean similarity index of our segmentation is 0.9200 for the left thalamus and 0.9239 for the right thalamus. The proposed method clearly leads to a higher mean similarity index compared to the five other methods, which provide similar overlap scores separately. The volumes measured by our method are closer to the manual expert segmentations than to the volumes measured by the five other methods. The results also show that there is no significant volume difference between segmented left and right thalamus.

**Table 2:** The Similarity Index (*SI*) on the MICCAI dataset (mean ± standard deviation) for the various segmentation methods as compared with manual segmentations.

Similarity Index (mean ± standard deviation)		
Method	Left Thalamus	Right Thalamus
STAPLE	0.9106 ± 0.0224	0.9132 ± 0.0229
Spatial STAPLE	0.9109 ± 0.0218	0.9136 ± 0.0222
Major Voting	0.9118 ± 0.0205	0.9126 ± 0.0239
Weight Voting	0.9119 ± 0.0204	0.9128 ± 0.0237
SIMPLE	0.9100 ± 0.0225	0.9141 ± 0.0210
Our	0.9200 ± 0.0124	0.9239 ± 0.0100

**Figure 6:**
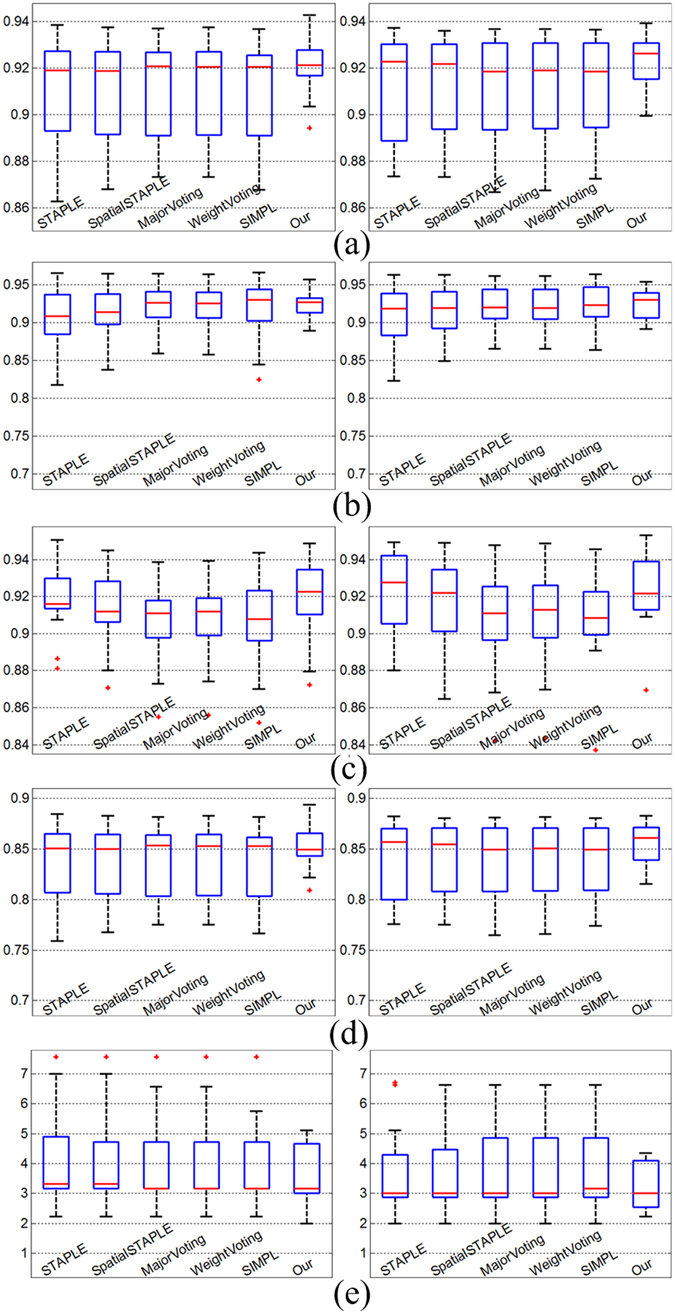
Box-plot of performance metric on the MICCAI dataset. The left column is left thalamus and the right column is right thalamus. (**a**) Similarity index (*SI*), higher values implying greater volumetric overlap with the manual segmentation, (**b**) Precision (*P*), (**c**) Recall (*R*), (**d**) Relative Overlap (*RO*) and (**e**) Hausdorff Distances (*HD*), lower values implying a surface closer to the manual segmentation.

Figure 6 shows summary box-plots of the similarity index (*SI*), precision (*P*), recall (*R*), relative overlap (*RO*), and Hausdorff distance (*HD*) versus the manual delineate for each segmentation method on the MICCAI dataset. The results show that our method using the level set fusion strategy is better than the state-of-the-art fusion algorithms.

**Figure 7:**
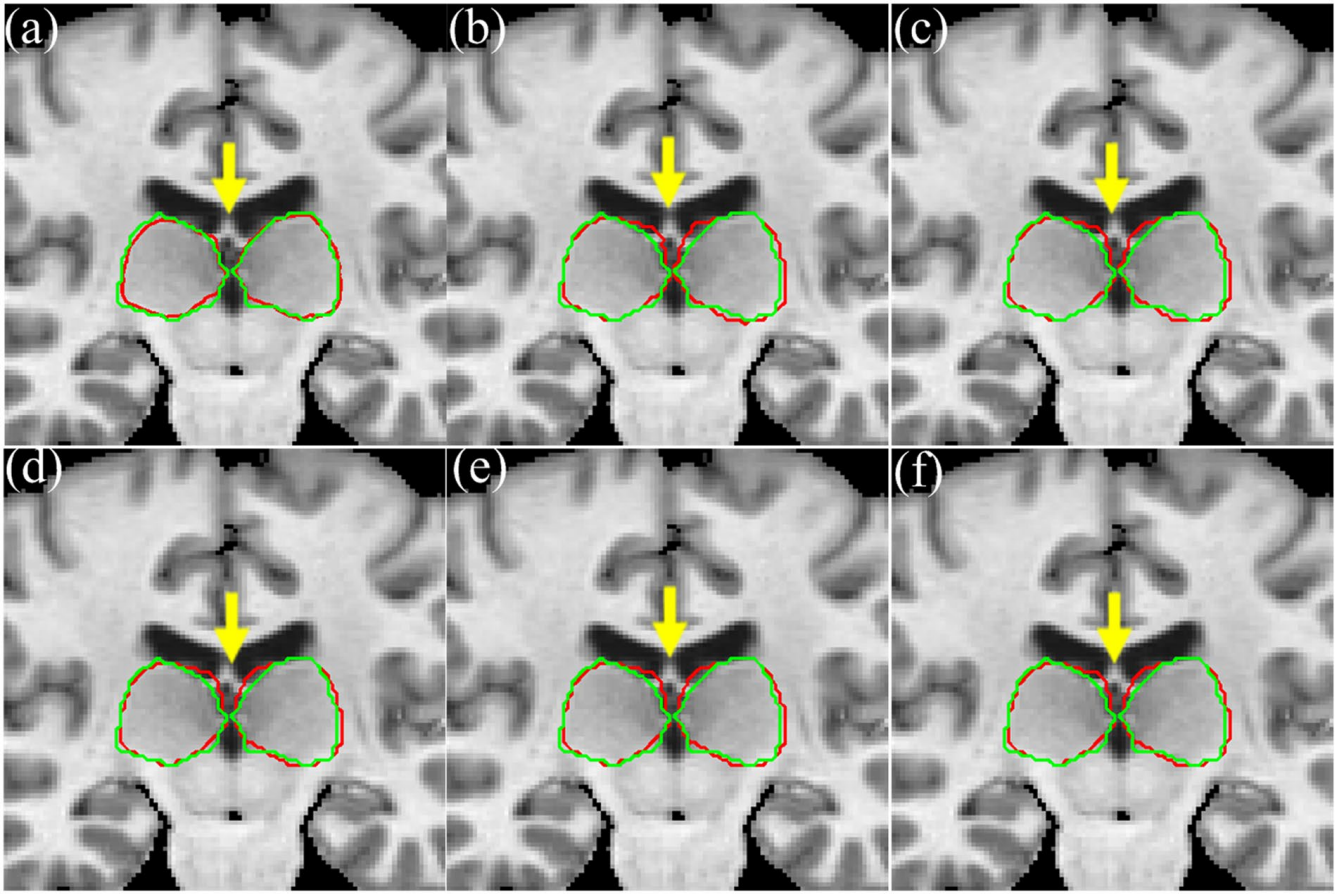
The segmentation results of MICCAI image No. 1006 by our method and five other methods. (**a**) Our method, (**b**) STAPLE, (**c**) Spatial STAPLE, (**d**) Major Voting, (**e**) Weight Voting, (**f**) SIMPLE. Automatic segmentation results are illustrated in red contour, the manually-delineated thalamus boundary shown by the green contour.

For visual comparison, we show in Fig. 7 the segmentation results of MICCAI image No.1006 by our method and five other methods, along with the manual segmentation. It can be observed that the thalamus segmented by our method is more similar to the ground-truth than any other method, e.g., STAPLE, Spatial STAPLE, Major Voting, Weight Voting, and SIMPLE. Particularly, in the region of the yellow arrow, the five other methods cannot locate the left and right thalamus. Our method can correct the registration errors, because the region-scalable fitting (RSF) model in the level set fusion framework can guide the motion of the contour toward the thalamus boundaries.

**Figure 8:**
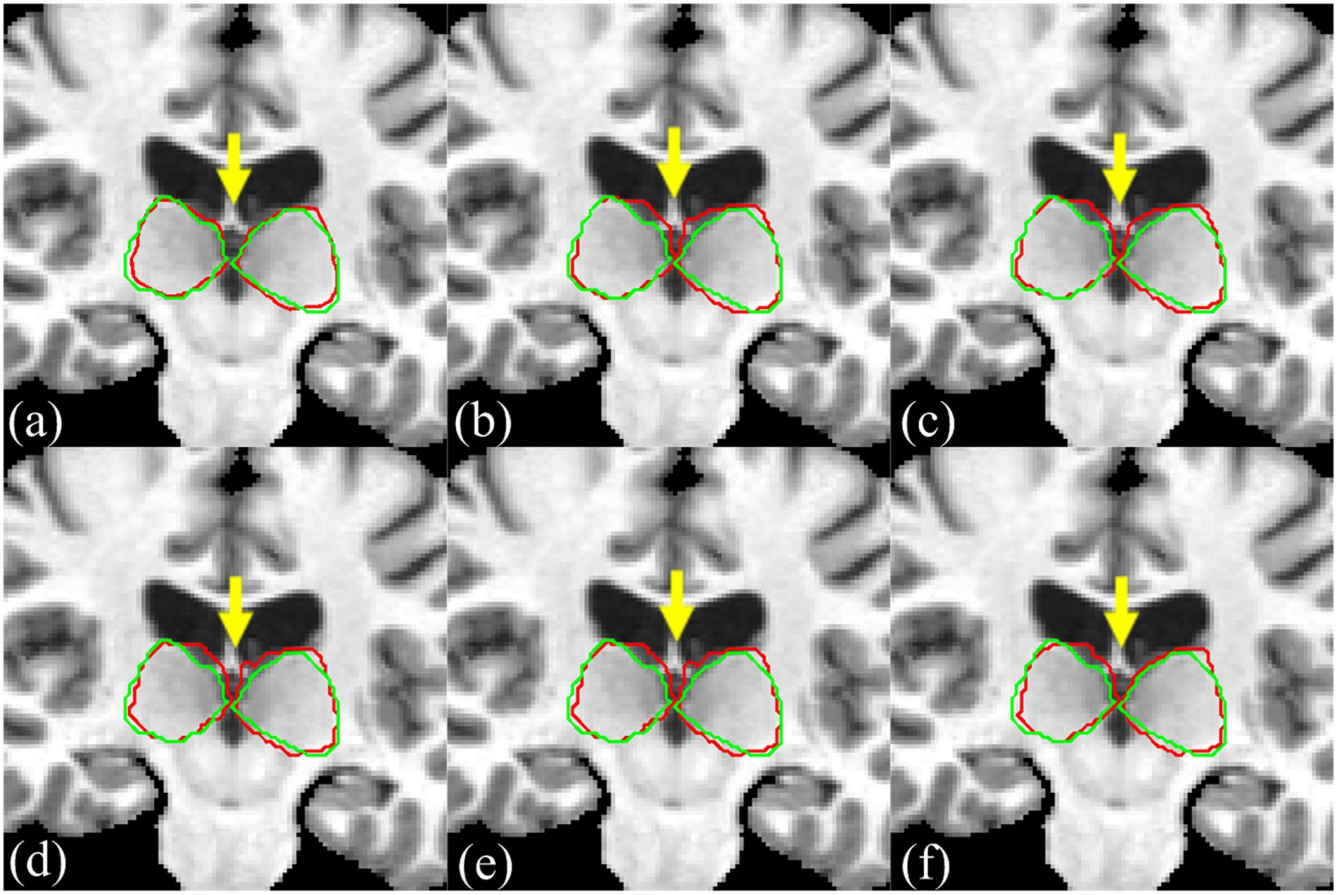
The segmentation results of MICCAI image No. 1025 by our method and five other methods. (**a**) Our method, (**b**) STAPLE, (**c**) Spatial STAPLE, (**d**) Major Voting, (**e**) Weight Voting, (**f**) SIMPLE. Automatic segmentation results are illustrated in red contour, the manually-delineated thalamus boundary shown by the green contour.

**Figure 9:**
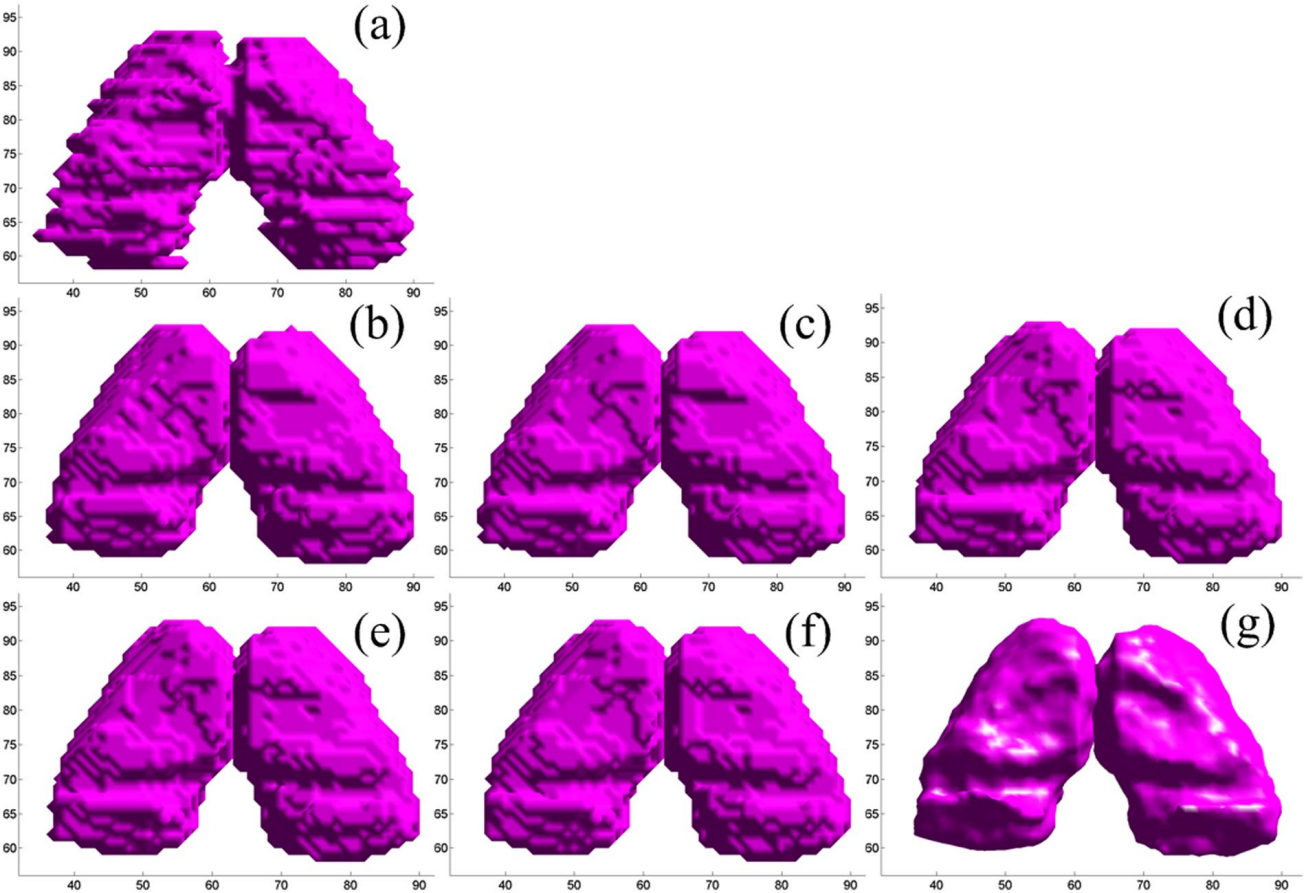
Surface rendering of ground truth and segmentation results (MICCAI, image No. 1006). (**a**) The manual segmentation, (**b**) STAPLE, (**c**) Spatial STAPLE, (**d**) Major Voting, (**e**) Weight Voting, (**f**) SIMPLE and (**g**) our proposed method.

We also show in Fig. 8 the segmentation results of MICCAI image No.1025 by our method and five other methods along with the manual segmentation. It can be clearly seen that the proposed method can accurately delineate the thalamus boundaries, especially for the regions near the lateral ventricle. The SIMPLE method can correct the registration errors in left thalamus as shown by the blue arrow, but it cannot correct the errors in right thalamus as shown by the yellow arrow. In addition to showing the two-dimensional slice of segmentation results, we present a three-dimensional visual inspection of the experimental results. In Fig. 9, we show the results of STAPLE, Spatial STAPLE, Major Voting, Weight Voting, SIMPLE and the proposed method. The five other methods are able to perform reasonable segmentations, but our method is able to provide smooth and closed contours on the final segmentation.

## Discussion

In this section important properties and possible alternative schemes of the proposed method are discussed. To the best of our knowledge, the proposed level set label fusion framework is new to the multi-atlas based segmentation. Our level set fusion framework is flexible and can be integrated with other types of registration methods and image based term for the possible improvement of thalamus segmentation. The choice of registration method will influence the overall performance of the segmentation the thalamus. One reason to use ANTS and DRAMMS as the registration methods in this paper is due to their topology preserving characteristic. Another reason is that both calculate the local similarity of each voxel in the registration procedure, and we can use the local similarity as the local weight when fusing the propagated labels.

The region-scalability of the image based term RSF is due to the kernel function with a scale parameter, which allows the use of intensity information in regions at a controllable scale, from small neighborhoods to the entire domain. As a result, this method can segment images with low contrast.

Currently, our method is implemented with Matlab 2012a on an Intel(R) Core(TM) 2.93 GHz CPU, the average time for segmenting a new image was approximately 2 min. However, our algorithm can be optimized and implemented using C/C++ in the future for significant improvement of the segmentation speed. The program can be further improved by using parallel programming techniques to reduce the segmentation time.

## Conclusion

The purpose of this work is to develop a new framework for the segmentation of the thalamus in magnetic resonance brain images. Segmentation of the thalamus is difficult and challenging because the structure boundaries may be blurry or even missing, and the surrounding background is full of irrelevant edges. To tackle these problems, we propose a novel method for segmenting the thalamus that combines multiple registration methods and a level set fusion strategy. The multiple registration methods are implemented by registering multiple atlas images to the target image using ANTS and DRAMMS. The incorporation of the level set fusion strategy enables the method to segment a thalamus with poor image contrast and to increase the robustness against errors in the registration procedure.

Multi-atlas segmentation is strongly dependent on the accuracy of registration. Using multiple registration methods can combine the complementary advantages of the different registration methods and can effectively reduce the registration errors. In the level set framework, the image based term guides the level set evolves toward the desired boundary of thalamus, the fusion term fuses the propagated labels and the regularization term maintains a regular shape. The level set formulation takes into account both the image information and the regularity of the thalamus. It can fuse the labels and correct registration errors simultaneously.

The proposed method has been evaluated on a brain MR image database consisting of thirty-five patients and was further compared with several state-of-the-art brain MR segmentation algorithms using various evaluation metrics. Experimental results demonstrate that: 1) our multi-method outperforms label fusion using single registration method, and 2) the proposed method consistently achieves higher segmentation accuracy than any of the other methods under comparison.

If there is no correspondence between the target images and the atlas images, no label is propagated. This prevents an incorrect label from being introduced into the estimation of the final segmentation. Thus, further work is needed to improve the robustness of this level set label fusion method to a large diversity of pathologies and anatomical structures, such as lesions or tumors and to use this technique to potentially detect these pathological patterns.
